# Prevalence of substance use amongst people living with human immunodeficiency virus who attend primary healthcare services in Mthatha, South Africa

**DOI:** 10.4102/safp.v62i1.5042

**Published:** 2020-06-04

**Authors:** Ramprakash Kaswa, Marietjie de Villiers

**Affiliations:** 1 Department of Family Medicine and Primary Care, Faculty of Medicine, Stellenbosch University, Cape Town, South Africa

**Keywords:** Alcohol, Smoking and Substance Involvement Screening Test questionnaire, ASSIST, HIV, primary healthcare, PHC, people living with HIV, PLWH, substance

## Abstract

**Background:**

About 13.3% of the South African population use some kind of substance during their lifetime. The incidence of substance use disorders is twice the global average. The use of various substances amongst people living with human immunodeficiency virus (PLWH) has increased tremendously in recent years. The growing culture of substance use amongst PLWH is a serious threat adding to the human immunodeficiency virus (HIV) epidemic and is likely to compromise the continuity of HIV care.

**Methods:**

A cross-sectional descriptive survey recruited adult PLWH who attended primary healthcare (PHC) services in Mthatha between 15 March and 15 April 2018. The Alcohol, Smoking and Substance Involvement Screening Test questionnaire (ASSIST), a tool validated by the World Health Organization, was used for data collection.

**Results:**

Out of a total 347 participants, 53% reported lifetime substance use and 32% admitted current use of a substance. Alcohol was the most common substance reported, followed by tobacco and cannabis. The majority of participants were female (75.2%), unemployed (70.8%), had secondary school-level education (85.2%) and a per capita household income below the national food poverty line (75%). The mean age of the participants was 37.9 years (standard deviation [SD] ± 10.33); this was marginally higher for male (39.9 years; SD ± 10.92) than female (37.2 years; SD ± 10.06) participants.

**Conclusion:**

This study has shown that there is a high prevalence of lifetime and current alcohol abuse amongst PLWH who make use of PHC services in the Mthatha area of South Africa. Of particular concern are the strong pointers towards younger people and males.

## Introduction

Globally, a quarter of a billion, or 1 in 20 people between the ages of 15 and 64 years, used a substance at least once in 2016.^1^ Substance abuse is widely defined as the harmful and hazardous use of tobacco, alcohol and psychotic substances.^2^ An estimated 31 million people who use substances suffer from substance dependency. The global estimated prevalence of substance use is 5.2% and the rate has been steady since 2011.^1^ About 0.6% of the global adult population suffers from substance dependency.^1,3^ Globally, cannabis (dagga) is the most commonly used substance, followed by amphetamine and opioids. Substance use and dependence have become a global concern.^3^

About 13.3% of the South African population uses at least one substance during their lifetime. The prevalence of substance use disorders is twice the global average. Alcohol is the most commonly used substance in South Africa, followed by tobacco and cannabis. About 7.06% of the population uses a narcotic substance at some stage in their lifetime and one in 14 uses it regularly. Inhalant substances are more popular amongst the youth and cannabis is the substance of choice, followed by methamphetamine (tik), amphetamine and heroin.^4,5^

Globally, the prevalence of substance use amongst people living with HIV (PLWH) is reported to be much higher than amongst the general population. In North America, an estimated 70% of PLWH practise substance use.^6,7^ Hartzler et al. reported that 48% of the PLWH population in the United States use at least one substance and 20% of them use multiple substances. Cannabis is the most common substance reported amongst PLWH, followed by alcohol and amphetamine.^8^

Substance use amongst PLWH is emerging as a health syndemic (coexistence of HIV and substance use). The substance use epidemic is associated with delayed HIV diagnosis and increased transmission risk behaviour. The coexistence of substance use and HIV has negative consequences on the continuity of HIV care.^9^ Substance use has a complex relationship with HIV and has a detrimental effect on transmission, linkage to care, adherence to treatment and retention in care. Despite widespread availability of antiretroviral therapy (ART), the PLWH who undertook substance use had increased morbidity and mortality from HIV- and non-HIV-related causes.^10^ Granich et al. reported that substance use blunts the effectiveness of the universal test and treat (UTT) approach for HIV transmission.^11^ Substance users amongst PLWH often fail to attend HIV care despite early diagnosis. Even after linkage to care, a poor adherence rate is reported amongst PLWH who use substances. The increasing trend of substance use is further compromising the HIV linkage to care and adherence to anti-retroviral therapy. Substance use amongst PLWH is associated with a high viral load that leads to an increased HIV transmission rate, as well as perceived poor quality of life.^12,13^

Primary healthcare (PHC) settings are the basic unit of healthcare service. It is an entry point for both communicable and non-communicable disease prevention, diagnosis and management. The PHC system is a gateway for integrated and comprehensive HIV care in South Africa.^2,14^ Recently, the UTT was rolled out through the PHC platform. The PHC is the first contact a person has with the health system where a range of health issues are addressed. The PHC settings provide an opportunity to address the substance use problem amongst PLWH. Screening of substance use at PHC could especially benefit the PLWH and enable them to make informed decisions on substance use management.^15^

Substance use is often under-diagnosed in PHC settings. Despite the substantial health and social impact of substance use on the continuity of HIV care, patients reporting to PHC settings are not consistently screened for substance use.^14,15^ Addressing substance use in the context of PHC settings could increase access to and retention of care. Primary prevention and early management of substance use have proven to have better outcomes than treating substance dependence.^16^ Because there is only a limited number of specialised health professionals and health facilities that manage substance dependency in South Africa, it is critical for PHC workers to identify and manage substance use early, at the level of PHC settings.

This study is part of a larger project with the overarching aim to evaluate the co-morbidity of HIV and substance use, and the response of PHC services to such patients in the Mthatha region of the Eastern Cape. This article reports on the prevalence of substance use amongst PLWH who attend PHC services in the Mthatha region of the Eastern Cape province. We specifically sought to determine the type of substances used by PHC users who are living with HIV.

## Methods

This cross-sectional descriptive survey was conducted in King Sabata Dalindyebo (KSD) sub-district municipality in the Eastern Cape province of South Africa. The estimated population of KSD is about 451 710 people living in 105 240 households.^17^ Mthatha is the main town in the KSD sub-district municipality. Most (98%) of the population are isiXhosa-speaking and rural. It is one of the poorest districts in which most people depend on social welfare grants and state facilities for healthcare services; only an estimated 4.6% have medical insurance.

The healthcare services in the KSD sub-district are rendered by one central and one regional hospital, as well as five community health centres (CHCs) and 42 clinics. The catchment areas of three of these CHCs are Mthatha township and the remaining two are located in the outskirts of the township. These CHC were divided into two strata, based on geographic location and the catchment areas of the population. One CHC from each stratum was selected for the current study, namely, Ngangelizwe and Mbekweni CHC. Ngangelizwe is the biggest CHC serving the township community, and Mbekweni is the biggest CHC serving an area on the outskirts of the township. Mbekweni is situated 20 km away from Mthatha.

### Sample size

The sample size was calculated with the help of a bio-statistician. A sample size of 350 produced a 95% confidence interval (CI) with a width equal to 10% when the sample proportion was assumed to be 30% of the PLWH. According to KSD sub-district monthly statistics, an average of 4000 PLWH visited the two selected CHCs every month. Researchers targeted 1 month to collect data after calculating the average number of PLWH visiting the selected PHCs.

### Study participants

**FIGURE 1:**
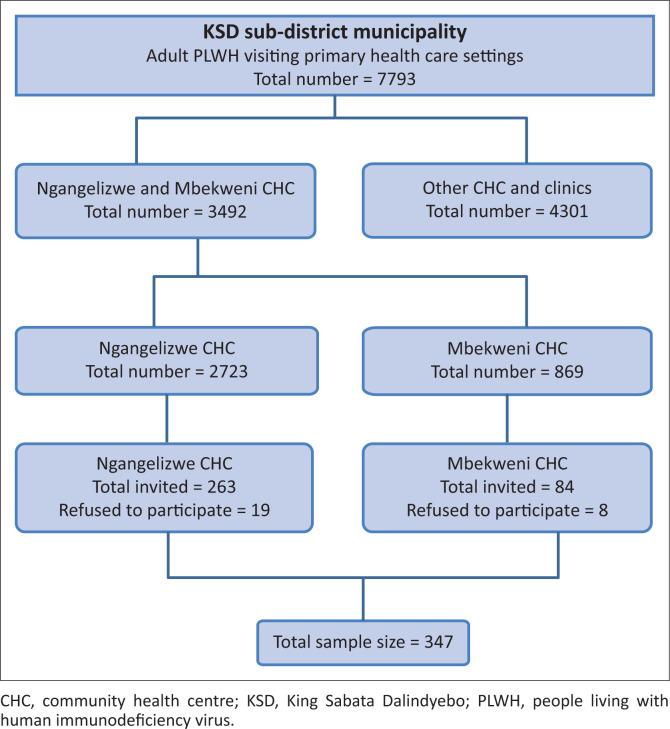
Sampling frame for people living with human immunodeficiency virus in King Sabata Dalindyebo sub-district municipality who visited primary healthcare settings between 15 March and 15 April 2018.

The entire study population consisted of adult (18 years and older) HIV patients who visited PHC settings in the KSD district municipality between 15 March and 15 April 2018. According to KSD sub-district statistics, the number of adult HIV patients who visited PHC settings during this period was 7793. During the same period, 3492 adults attended HIV clinics at the two selected CHCs. A simple random sampling method was used to identify participants. Ten participants were selected from a list of 1 to 100 in order of arrival at the HIV clinic, using random numbers generated in an Excel spreadsheet from each CHC on a particular day. If a patient refused to participate, the immediate next number was used. Critically ill patients were excluded from the study. Participants who met the inclusion criteria were approached and invited to participate after giving written informed consent. The sampling frame for the current study is demonstrated in Figure 1 as a flow chart.

### Data collection and analysis

Trained research assistants administered a structured questionnaire to collect demographic data on age, gender, household income, cluster of differentiation 4 [CD4] and viral load. Substance use, type, duration and severity were measured by using the Alcohol, Smoking and Substance Involvement Screening Test (ASSIST) validated by the World Health Organization (WHO).^18^ It is a screening tool used for the measurement of tobacco, alcohol and other psychoactive substances used during the lifetime and in the past 3 months in PHC settings. The tool also measures the degree of substance use and dependence. The tool comprises eight questions. The first question asks about lifetime use of the following 10 commonly available substances: tobacco, alcohol, cannabis, cocaine, amphetamine-type stimulants, inhalants, sedatives, hallucinogens, opioids and other drugs. If the participant responds negatively to all substances in the first question, the interview is terminated. In case of endorsement of one or more substances during the lifetime, the participant proceeds to questions 2–5. These questions ask about the frequency of use, craving and frequency of health, social, legal or financial problems related to recent substance use. Questions 6 and 7 ask about lifetime substance-related problems (expressed concern about substance use, failed attempt to control, failure to meet the expected obligation). Each question is rated from 0 for never to 5 points for daily use. Question 8 enquired about intravenous drug use. A total risk score calculated from the completed ASSIST interview was based on the sum of scores from questions 2–7. A risk category was assigned, indicating the risk for each substance queried (for alcohol: low = 0–10; moderate =11–26; high = 27+; for other substances: low = 0–3; moderate = 4–26, high = 27+). The ASSIST risk categories determine the substance-related risk and the most appropriate intervention.^15,16^ The tool is validated for use in PHC settings and has previously been used in the South African context.

Two research assistants were recruited from outside the study community because of the sensitivity and stigma associated with substance use disclosed during the interview. The research assistants were retired nurses and fluent in both English and IsiXhosa. The research assistants attended a simulated patient training workshop for adoption of the standard procedure of the ASSIST tool. The data were collected simultaneously from both health centres. All interviews were conducted in English language in a private consulting room to prevent any interruption and to ensure privacy.

Each participant was assessed using the ASSIST tool, after obtaining written informed consent from participants. Data were entered on an Excel spreadsheet and every10th item on the sheet was double-checked for correctness. The data were analysed by biostatisticians. Statistical Package for Social Sciences (SPSS) version 18 was used. Frequency analysis was used to determine lifetime substances use as well as use during the past 3 months. The ASSIST risk score was calculated as described above and as a standard procedure for the ASSIST. Cross-tabulation between substance use (lifetime and past 3 months) and other demographic characteristics was analysed. The Pearson Chi-squared test was used for categorical data to evaluate any significant difference in the (*p* < 0.05) level observed.

### Ethical considerations

The Health Research Ethics Committee (HREC) of Stellenbosch University approved the study (HREC reference number: S18/01/001). The study was also approved by the Department of Health, Eastern Cape (National Health Research Database [NHRD] reference number EC_201803_007), as well as the local health authorities.

## Results

**FIGURE 2:**
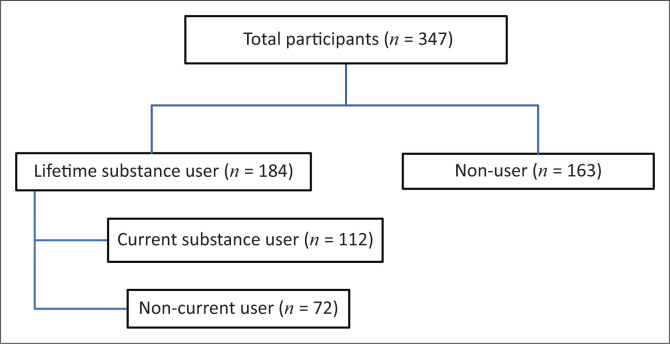
The distribution of participants in the current study.

There were 347 PLWH (Figure 2) who participated in the study. The mean age was 37.9 years (standard deviation [SD] ± 10.33). This was slightly higher amongst male (39.9 years; SD ± 10.92) than amongst female (37.2 years; SD ± 10.06) participants. The majority of participants were female (75.2%), unemployed (70.8%) and had secondary school-level education (85.2%) and a per capita household income below the national food poverty line (75%). About two-thirds of participants disclosed their HIV status to close family members. Table 1 presents the demographic and clinical characteristics of lifetime and current substance use amongst PLWH.

**TABLE 1:** Demographic and clinical characteristics of lifetime and current substance use amongst people living with HIV in Mthatha, South Africa (*n* = 347).

Variable	Total population	Lifetime substance use				Substance use in the last 3 months			
		*n*	%	*χ* ^2^	*p*	*n*	%	*χ* ^2^	*p*
**Age (years) (*n* = 347)**	-	-	-	18.3	< 0.001*	-	-	11.0	0.012*
18–30	88	62	67.4	-	-	41	44.6	-	-
31–40	129	69	53.5	-	-	41	31.8	-	-
41–50	87	31	35.6	-	-	19	21.8	-	-
Above 50	39	22	56.4	-	-	11	28.2	-	-
Total	-	184	-	-	-	112	-	-	-
**Gender (*n* = 347)**	-	-	**-**	57.3	< 0.001*	**-**	**-**	56.4	< 0.001*
Male	86	76	84.4	-	-	56	65.1	-	-
Female	261	108	41.4	-	-	56	21.5	-	-
Total	-	184	-	-	-	112	-	-	-
**Occupation (*n* = 347)**	-	-	-	0.017	0.895	-	-	0.163	0.393
Employed	101	53	52.5	-	-	31	30.7	-	-
Unemployed	246	131	53.3	-	-	81	32.9	-	-
Total	-	184	-	-	-	112	-	-	-
**Education status (*n* = 345)**	-	-	-	2.6	0.268	-	-	0.676	0.713
Primary school	35	23	65.7	-	-	10	28.6	-	-
High school	294	151	51.4	-	-	96	33.0	-	-
Degree or diploma	16	8	50.0	-	-	4	25.0	-	-
Total	-	182	-	-	-	110	-	-	-
**Per capita household income (*n* = 344)**	**-**	-	-	0.522	0.770	-	-	0.411	0.814
Less than R532.00†	258	140	54.3	-	-	84	32.6	-	-
R532 to R1138.00‡	31	15	48.4	-	-	11	35.5	-	-
> R1138.00	55	28	50.9	-	-	16	29.1	-	-
Total	-	183	-	-	-	110	-	-	-
**Disclosure of HIV (*n* = 328)**	**-**	**-**	**-**	2.07	0.557	**-**	**-**	6.33	0.097
Friends	19	9	47.4	-	-	7	36.8	-	-
Life partner	78	45	57.7	-	-	31	39.7	-	-
Family member	231	118	51.3	-	-	63	27.8	-	-
Total	-	173	-	-	-	102	-	-	-
**CD4 counts (*n* = 292)**	-	-	**-**	1.3	0.86	-	**-**	8.3	0.08
Less than 50	21	12	57.1	-	-	6	28.6	-	-
50–100	19	8	42.1	-	-	6	31.6	-	-
101–200	40	19	47.5	-	-	4	10.0	-	-
201–500	100	53	53.0	-	-	33	33.0	-	-
More than 500	112	56	50.0	-	-	32	28.6	-	-
Total	-	142	-	-	-	81	-	-	-
**Viral load (*n* = 106)**	**-**	**-**	**-**	0.123	0.726	**-**	**-**	0.005	0.946
Detectable	80	43	53.8	-	-	21	26.3	-	-
Non-detectable	26	15	57.7	-	-	7	26.9	-	-
Total	-	58	-	-	-	28	-	-	-

CD4, cluster of differentiation 4.

* , *p* < 0.05 statistically significant.

† , R532 food poverty line (Stat SA 2017).

‡ , R1138 upper bound of the poverty line (Stat SA 2017).

The prevalence of lifetime and current substance use amongst PLWH was 53% (95% CI: 47.63 – 58.36) and 32.3% (95% CI: 27.44 – 37.52), respectively. The lifetime substance used most often was alcohol (47.8%), followed by tobacco (29.7%), cannabis (5.7%) and other substances (2.9%). The highest prevalence of substance use occurred in the 18–30-year-old age group. The prevalence of substance use decreased with advancing age and a statistically significant difference was observed with different age group participants (lifetime user *χ*^2^ = 18.3, *p* = 0.001, current user *χ*^2^ = 11.0, *p* = 0.012).

The prevalence of lifetime substance uses amongst male (84%) participants is twice as high as amongst their female (41%) counterparts (*χ*^2^ = 57.3, *p* < 0.001). The prevalence of current substance uses amongst male (65%) participants is three times higher than that of their female (21%) counterparts (*χ*^*2*^ = 56.4, *p* < 0.001). The majority of PLWH had a CD4 count of more than 200 and there was no statistically significant difference between substance use and CD4 counts.

**FIGURE 3:**
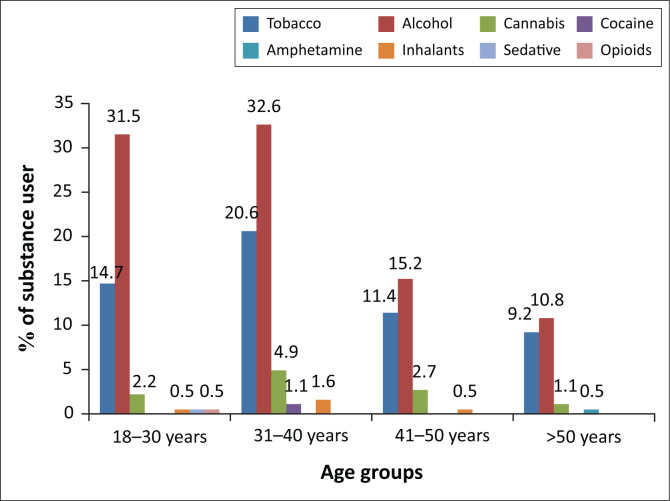
Lifetime use of substances amongst people living with human immunodeficiency virus in Mthatha, South Africa (*n* = 184).

The prevalence of different substances used by PLWH according to their age category is demonstrated in Figure 3. Alcohol was the most commonly used substance amongst PLWH in all age categories, followed by tobacco, cannabis and other substances.

Table 2 presents the ASSIST risk score for different substances used by PLWH. The mean risk score for tobacco users (15.4 ± 7.9) was slightly higher than that for alcohol users (12.1 ± 7.8). About half (55%) of alcohol users and four-fifths (81%) of tobacco users scored in the moderate risk category. Only seven substance users (ASSIST risk score >26) were classified as being in the high-risk category of substance use, namely, three alcohol users, three tobacco users and one cocaine user.

**TABLE 2:** Alcohol, Smoking and Substance Involvement Screening Test risk score of current substance use amongst people living with human immunodeficiency virus in Mthatha, South Africa.

Variables	ASSIST				ASSIST risk score current use		ASSIST risk level of current use†						
	Lifetime use		Current use		Mean score	SD	Low		Moderate		High		
	*n*	%	*n*	%			*n*	%	*n*	%	*n*		%
Alcohol	166	47.8	96	27.6	12.1	7.88	40	41.6	53	55.2	3	3.1	
Tobacco	103	29.7	65	18.7	15.4	7.93	9	13.8	53	81.5	3	4.6	
Other substances‡	30	8.6	4	1.1	20.75	13.93	1	25	2	50	1	25	

ASSIST, Alcohol, Smoking and Substance Involvement Screening Test; SD, standard deviation.

† , The risk level for alcohol: low = 0–10, moderate = 11–26, high = more than 26; for other substances: low = 0–3, moderate = 4–26, high = more than 26.

‡ , Other substances included cannabis, cocaine, amphetamine, opioids and sedatives.

## Discussion

Alcohol is the most commonly used substance reported amongst PLWH in PHC facilities. The data reported here document a high lifetime and current prevalence of alcohol abuse. These findings are consistent with a countrywide estimation of substance use and are consistent with the use reported in other sub-Saharan countries.^5,19^ South Africa has one of the highest per capita alcohol consumption rates in the world. Although many studies reported geographical differences in the prevalence of substance use, alcohol remains the substance used most often by South Africans.^20,21^

The lifetime prevalence of alcohol use (47.8%) amongst PLWH in this study is much higher than that amongst the general population (38.7%) as reported by a South African stress and health study.^4,22^ Similar alcohol consumption amongst PLWH was reported in many African countries, such as Zambia (44.3%), Tongo (40.6%) and Côte d’Ivoire (46.4%).^5,20^ Some studies even demonstrate that the alcohol consumption rates amongst PLWH are almost twice as high as amongst the general population.^21^ Although alcohol consumption is widespread in the general population, hazardous use is much higher amongst PLWH, resulting in decreased overall life expectancy in this group.^5^ The psychological impact of the dual stigma of HIV infection and alcohol consumption hinder health-seeking behaviour for people who are both HIV-positive and substance users.^23^ Substance users amongst PLWH often fail to attend HIV care despite early diagnosis. Even after linkage to care, a poor adherence rate is reported amongst PLWH who use substances.

It is notable that the proportion of lifetime and current substance use was high amongst younger participants than their older counterparts but the findings from the current study could not establish the age of substance use initiation. The national and international research has reported that substance use generally commences at a younger age.^14,24^ Adolescence is a critical period of physical and psychological development when substance use problems and other risk-taking behaviour commence. There are many reasons for use of substances amongst adolescents, including desire of a new experience, peer pressure, stress and an attempt to deal with problem. Substance use is generally initiated around the age of 11 years or sometimes even at a younger age. The exact age for substance use screening nevertheless depends on the local prevalence and pattern of substance use.^25^ Because the study demonstrated a high prevalence of substance use amongst PLWH, it is appropriate to commence regular screening at PHC facilities.

The findings reported heavy alcohol consumption amongst males compared with their female counterparts. Higher consumption of alcohol amongst males has been reported both in sub-Saharan Africa and in developed countries in North America and Europe.^26^ It is attributed to the acceptance of male drinking as part of the social and masculine norm in many societies, whilst the same societies do not apply these norms for women. Overall, substance use tends to be higher amongst males than amongst females. These findings are consistent with reported research in sub-Saharan Africa.^27,28^

The majority of PLWH who consume alcohol and other substances are unemployed and living in a household with a per capita income below the food poverty line (R532.00 per capita). Alcohol product in South Africa is more affordable than in most low- and middle-income countries.^29^ This might be a possible explanation of alcohol abuse even in people of low socio-economic status. Similar results were reported in a study based on nationally representative data from South Africa, which stated that the harmful use of alcohol amongst PLWH was much higher amongst people of low socio-economic status. In contrast, Probst et al. reported that there was no difference in consumption of alcohol amongst PLWH in different socio-economic classes in South Africa, but high HIV-associated mortality and poor clinical outcomes were attributed to low socio-economic status amongst PLWH.^30^ Furthermore, it states that affordability of alcohol products is an important determinant of demand and increase in taxes and prices can limit the consumption and demand of alcoholic products.^29^

The majority of substance users amongst PLWH have a low-to-moderate (score < 27) risk, according to the ASSIST tools. Primary care workers therefore have an ideal opportunity for early intervention before serious substance dependency develops. Many research findings support the notion that low-to-moderate risk of substance use has shown significant improvement when simple motivational interviewing techniques are used.^31^ Furthermore, it is stated that this brief intervention may easily be integrated within context-specific PHC settings. In particular, brief intervention for alcohol use has emerged as a cost-effective and practical approach for PHC settings. These interventions are particularly short in duration and promote positive behavioural changes. Although brief intervention is often not feasible as a treatment on its own for high-risk patients, it provides the foundation for PHC workers for detailed clinical assessments and appropriate specialist referrals.^18,21^

### Limitations of the study

The researchers acknowledge several limitations. The information on substance use was self-reported, which may have compromised its reliability in terms of over-reporting or under-reporting. The study did not use any biomarker or collateral information from family members to verify the use of the substance. Furthermore, the sample is derived from PHC users in a local context and in a selected population of PLWH.

## Conclusion

This study has shown that there is a high prevalence of lifetime and current alcohol abuse amongst PLWH who make use of PHC services in the Mthatha area of South Africa. Of particular concern are the strong pointers towards younger people and males. The age and gender-sensitive policy interventions would need to address these challenges if we would like to reduce the effects of these results on the HIV epidemic in the Mthatha region. Given the high prevalence of substance use amongst PLWH in primary care users indicates the needs of routine screening substance use.
